# Progress in Advanced Properties of Electrowetting Displays

**DOI:** 10.3390/mi12020206

**Published:** 2021-02-18

**Authors:** Yi Lu, Biao Tang, Guisong Yang, Yuanyuan Guo, Linwei Liu, Alex Henzen

**Affiliations:** 1 Guangdong Provincial Key Laboratory of Optical Information Materials and Technology & Institute of Electronic Paper Displays, South China Academy of Advanced Optoelectronics, South China Normal University, Guangzhou 510006, China; yilu@m.scnu.edu.cn (Y.L.); biao.tang@guohua-oet.com (B.T.); linwei.liu@guohua-oet.com (L.L.); 2 National Center for International Research on Green Optoelectronics, South China Normal University, Guangzhou 510006, China; 3 Academy of Shenzhen Guohua Optoelectronics, Shenzhen 518110, China; yuanyuan.guo@guohua-oet.com; 4 Shenzhen Guohua Optoelectronics Tech. Co. Ltd., Shenzhen 518110, China

**Keywords:** electrowetting display, technical developments, dielectric breakdown, bi-stable, optical stability

## Abstract

Electrowetting display (EWD) has promising prospects in the electronic paper industry due to it having superior characteristics, such as the ability to provide a comfortable reading experience and quick response. However, in real applications, there are also problems related to dielectric deterioration, excess power consumption, optical instability and narrow color gamut etc. This paper reviewed the existing challenges and recent progress made in terms of improving the optical performance and reliability of EWD. First, the principle of electrowetting applied in small and confined configurations is introduced and the cause of the failure of the dielectric layer is analyzed. Then, the function of the pixel structures is described to avoid display defects. Next, electric signal modulations are compared in terms of achieving good image quality and optical stability. Lastly, the methods are presented for color panel realization. It was concluded that multi-layer dielectrics, three-dimensional pixel structures, proper electric frequency-and-amplitude modulation and an RGB color panel are expected to resolve the current limitations and contribute to designing advanced reflective displays.

## 1. Introduction

Electrowetting is the phenomenon of modulating surface wettability via an external electric field, which has drawn great attention in lab-on-a-chip microfluidics [1]. Electrowetting display (EWD) uses electrowetting to control the state of oil inks and display desired images, which has been at the center of attention since 2003, when Hayes and Feenstra reported on the possibilities contained in the system [2]. The road since then has been long and arduous. A plethora of challenges have required solving one by one. Research was later focused on the prediction of the working principle, dielectric material preparation, pixel structure design, electric signal modulation and color panel realization in order to achieve market applications and mass production.

In display theory, the basic principle of electrowetting has been demonstrated to apply in confined pixels. The surface wettability change between the hydrophobic (oleophilic) state and hydrophilic (oleophobic) state causes a switch between the “on” and “off” status in each pixel unit. Tang et al. [3] illuminated the mechanism of oil film opening and predicted the threshold voltage based on the electro-capillary instability of the oil–water interface in the pixel. Zhou et al. [4] formulated a theoretical model of dynamic electrowetting display to predict the opening rate of oil film.

Researchers have also dedicated efforts to the development of robust dielectric materials, as the deterioration of the dielectric layer will directly lead to the failure of the display device and greatly reduce the service time. The current preparation of dielectric layers is a lengthy and error-prone process which includes sample cleaning, film coating, surface treatment and photolithographic patterning [5]. Chen et al. [6] reported the advantages of screen printing techniques for preparing uniform and compact dielectric materials as compared with the conventional spin coating process. Guo et al. [7] selectively injected a fluoropolymer to form hydrophobic patterns based on a combined approach of inkjet printing and phase change filling. In this way, changes to the material properties during the surface treatment of reactive ion etching were avoided. These technical developments enabled the fast preparation of uniform and compatible multi-layer dielectrics.

A well-designed shape and structure of pixel guide the flows of oil inks in the confined system which resolve display defects. Early studies mainly focused on the planar surfaces by varying the shape and size of the pixel to restrict the fluid flow in the display unit [8]. Various shapes of the pixel, including quadrilateral, rectangle, triangle and trapezoid shapes were designed and the response rates and brightness of the images produced were compared. The quadrilateral pixel has a better response rate and brightness, but the improvement is still limited. Additionally, the planar surface still relies on the electric signal, while displaying images in case of backflows [9]. For a limited pixel size and spacing, overflows of the oil inks [10] to the adjacent pixels may take place. Three-dimensional structures reserve excess oil inks and prevent oil backflows and overflows, while the bi-stable features only require electric energy during image conversion, thus, enabling them to be used for a longer time [11]. In addition, “symmetry breaking structure” [12] on the original planar surface realizes the breaking of the oil film under a low voltage and ensures the oil ink flows in a uniform direction. 

For application of the electric signal, a proper driving waveform can not only achieve a good optical performance, but also improves the stability of the gray level [13]. The display performance refers to the image quality (e.g., reflectivity, brightness, resolution). Optical stability means that the generated picture will not fluctuate in a certain period of time. The optical performance and stability of EWD are mainly dependent on the electro-fluidic behaviors of oil inks in the pixel. Generally, a higher opening rate and larger aperture ratio are achieved by a larger driving voltage. However, a large voltage may cause oil splitting [14,15]. Conversely, lower voltages can ensure more stable oil ink flows and prevent the breakdown of the dielectric layer. Therefore, the selection of electric voltage requires a tradeoff between performance and stability. A proper electric driving scheme can achieve both expectations in the real operation. By using frequency-and-amplitude modulation and converting a constant voltage to several segments, Yi et al. [16] achieved both high levels of brightness and optical stability and also improved the response speed of oil inks. 

Current challenges for electrowetting displays are mainly related to dielectric deterioration, display defects, energy consumption in mono-stable states and color panel realization. In the following sections, the electrowetting principles in the confined configuration will be introduced. The failure mode of the dielectric layer will be analyzed in more details. Suitable pixel structures and driving schemes will be discussed to achieve high performance, optical stability and energy saving displays. Lastly, the latest status and highlights of R&D will be elaborated on in this article, providing the latest insights in the behavior of oil films in pixels, and the creation of colorful images.

## 2. Principles: Electrowetting in Confined Systems

Electrowetting produces its active influence by changing surface wettability using an external electric field. This effect can be used to control the contact angle of the electrolyte on a solid surface, which was first described in Young–Lippmann’s equation [17].

(1) $$\cos\theta =\cos\theta_{0}+\frac{CV^{2}}{2\sigma }$$

where $\theta_{0}$ is the initial contact angle, θ is the contact angle after applying electrowetting. C (=$\frac{\;\varepsilon_{0}\varepsilon_{r}}{d}$) is the capacitance. $\varepsilon_{0}$ is the vacuum permittivity. $\varepsilon_{r}$ and d are the relative permittivity and thickness of the dielectric layer, respectively. σ is the interfacial tension between the liquid and vapor and *V* is the applied voltage. For a large capacitance material, the contact angle can be greatly reduced via a relatively small voltage, which results in a surface wettability change from hydrophobic (oleophilic) to hydrophilic (oleophobic). 

**Figure 1:**
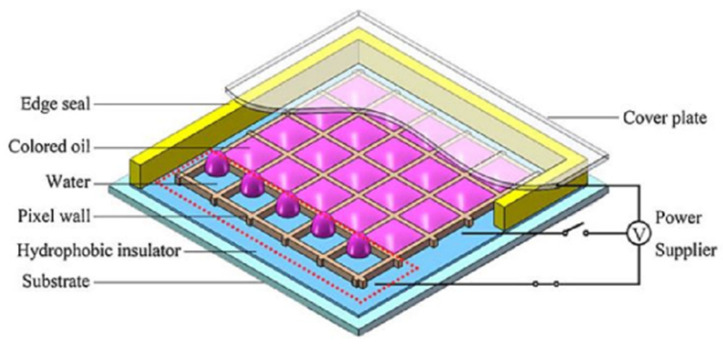
Schematic plot of electrowetting display. (Images are reproduced from reference [6] with the permission of Wiley-VCH Verlag).

**Figure 2:**
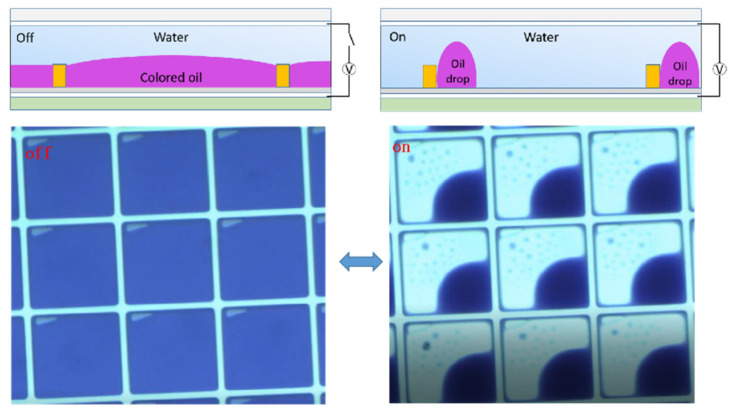
Experimental demonstration to show “on” and “off” state of electrowetting display (EWD) display units under electrowetting.

In electrowetting displays, two types of fluid exist in the confined unit; electrically insulating oil inks and electrically conductive liquid electrolytes (e.g., DI water) in the surrounding environment. Surface wettability plays a crucial role in the process of modulating the state of oil inks in order to change the aperture ratio of the pixel. A typical unit of the pixel is schematically shown in Figure 1. In Figure 2, the oil ink is initially filled in and completely covers each pixel unit. Once the electric signal is applied, the oil film recoils and an aqueous phase occupies the open section of the pixel. The electro-capillary instability was considered in the initial opening of oil films in electro-fluidic displays [3]. It was derived from the pressure balance on the oil-and-water interface, including the capillary pressure to maintain a flat interface and the electric stress to undulate the interface. p(r,t) represents the total force pressure considering a reference pressure of $p_{0}$.

(2) $$p\left(x,y,t\right)=\left(-\sigma {𝛻}^{2}+\frac{1}{2}C^{\prime \prime }\left(h\right)V^{2}\right)\zeta \left(x,y,t\right)+p_{0}$$

**Figure 3:**
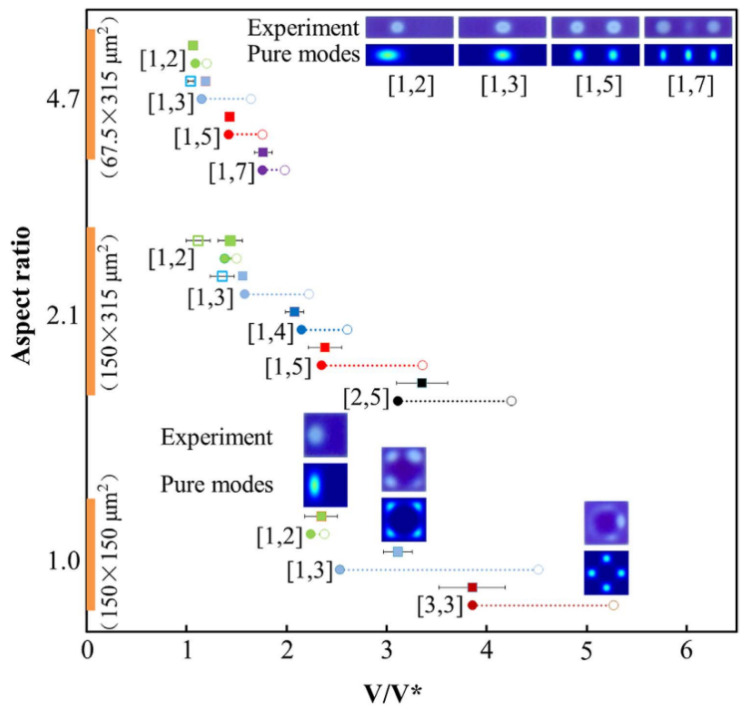
The experimentally observed wave modes and the theoretically expected sequences are compared in solid squares (the aspect ratio is defined as the ratio of width to the height of a pixel). (Images are reproduced from reference [3] with the permission of Springer Nature).

A positive value of this pressure promotes film growth. ζ(x,y,t) is the real-time interfacial location. $C^{\prime \prime }\left(h\right)$ is the second derivative of the capacitance of the oil film with an undisturbed thickness of *h.* In Figure 3, it is theoretically shown that the applied electric signal leads to the generation of a cascade of voltage dependent wave modes. A large voltage or a small oil film thickness will make the interface unstable, and consequently lead to the rupture of the oil film. In the course of validating the theory, it was experimentally observed that a number of undulation modes appear prior to film rupture, and the rupture location does not correspond to the maximum electric field strength in the case of the standard convex water/oil interface used in working devices. These findings provided an improved understanding of the dynamics of the oil rupture process inside the pixel after applying a voltage.

**Figure 4:**
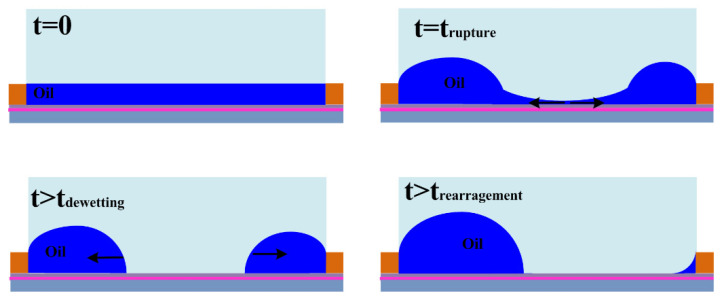
Stages in the on switching of an EWD pixel. (Images are reproduced from reference [4] with the permission of MDPI).

**Figure 5:**
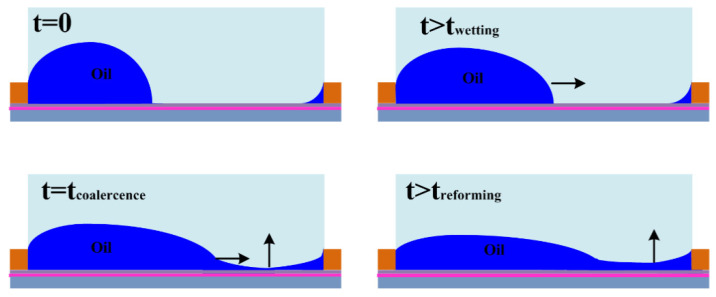
Stages in the off-switching process. (Images are reproduced from reference [4] with the permission of Elsevier).

Furthermore, the dynamic behaviors of oil inks in the dewetting and rewetting processes, corresponding to the “on” and “off” switching of the pixel unit, were analyzed in an electro-fluidic pixel [4]. For “on” switching, the oil motion leads to oil film rupture (initiation stage), oil-dewetting and a slower droplet rearrangement stage (Figure 4). For “off” switching, fast oil wetting and reforming of the surface to the flat (dark) state (Figure 5) occur. A dynamic model derived from the energy balance perspective has been employed to describe the electro-fluidic response and corresponding optical performance inside an electro-fluidic based display (EFD) pixel. The variability of oil ink motion between on and off-switching and the optical response delay during the on-switching process have been well described and addressed.

According to the theoretical approach of static and dynamic conditions for oil ink in confined pixels, this provided a straightforward approach to describe the complex electro-fluidic switching dynamics under electrowetting modulation, which may guide the further optimization of EFD device design and driving schemes. 

## 3. Materials: Multilayer Dielectric

**Figure 6:**
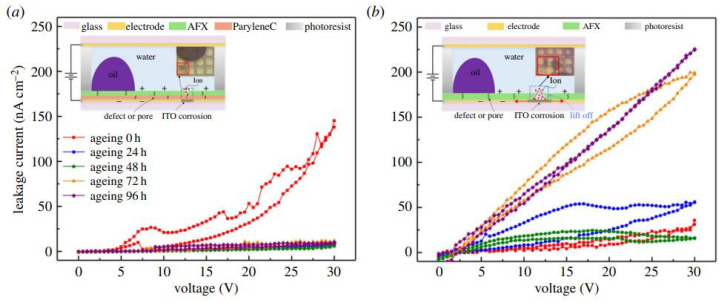
Leakage current measurements with stepwise applied voltage at different thermal aging testing conditions for (**a**) bilayer AFX (Teflon® AF series)/ParyleneC. (**b**) Single AFX (AFX: 1359 nm) device. The corresponding microscopy picture of the damage is given. (Images are reproduced from reference [18] with the permission of the Royal Society).

Dong et al. [18] fabricated a Parylene C and AF 1600 double layer structure to prevent dielectric breakdown. The advantages of a double layered dielectric material in terms of stability and reliability over a single layer dielectric material were evaluated by measuring the leaking electric charge. Zhou et al. further characterized the service time by comparing three different dielectric materials (Cytop (AGC), Teflon (Chemours) and Hyflon (Solvay)) through thermal aging testing [19]. The size and shape of the defects were measured under a scanning electron microscope, as shown in Figure 6, and the failure modes were analyzed in the development of manufacturing techniques. The pinhole-free and multi-layer structure can effectively avoid dielectric breakdown, therefore, this largely extends the reliability of the device. 

## 4. Pixel Architecture: Three-Dimensional Structures

### 4.1. Symmetry Breaking Structure

**Figure 7:**
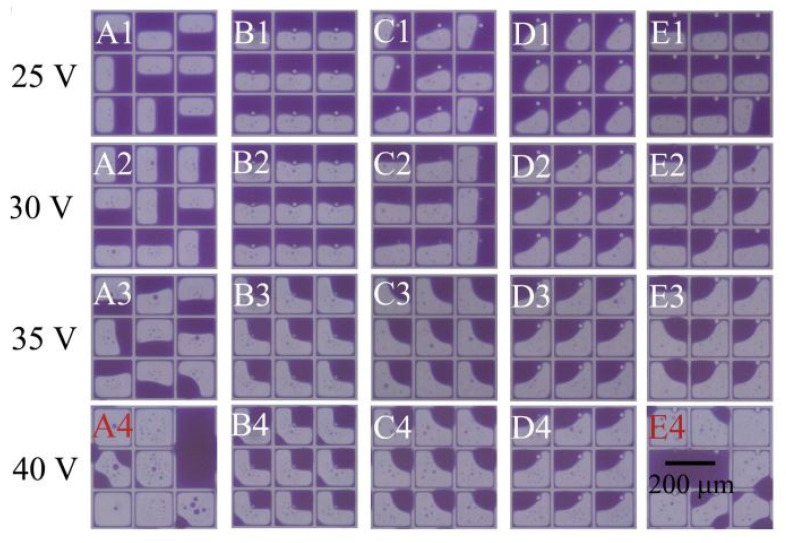
Symmetry breaking structure (EPS) in different pixel locations results in various oil opening shapes. (Images are reproduced from reference [12] with the permission of Elsevier).

A “symmetry breaking structure” in the pixel facilitates directional openings to achieve image/pixel symmetry consistency. The design of a symmetry breaking structure also increases the local electric field so that the oil film opens near to or above the symmetry breaking structure. The effects of the first effect, breaking the symmetry of the square pixel, thus determine the location of the oil film rupture and effectively guide the ink movement to a specific place inside the pixel, as can be seen in Figure 7 [12].

### 4.2. Oil Reserve Structure

**Figure 8:**
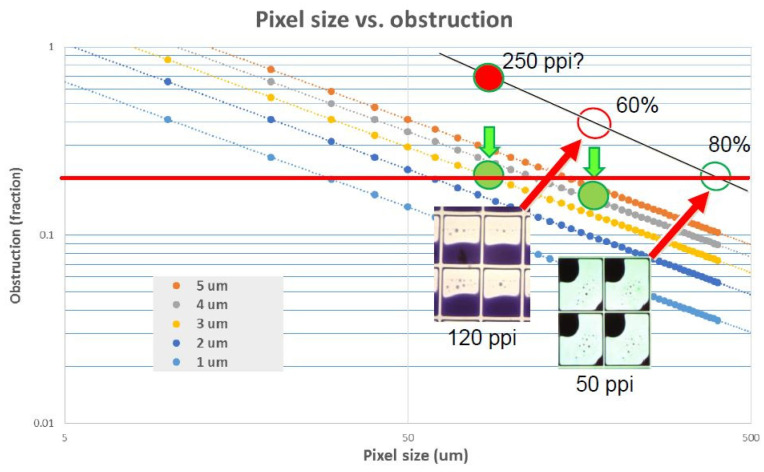
Relationship between aperture ratio, pixel size and oil film thickness (Images are reproduced from reference [20]).

For the conventional planar display unit, the size of the pixel is determined by the oil film thickness. Figure 8 shows a set of curves for the relationship between aperture ratio, pixel size and oil film thickness [20].

**Figure 9:**
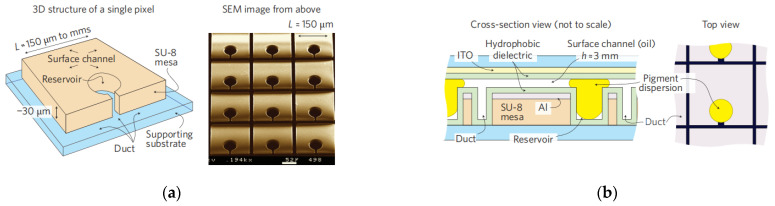

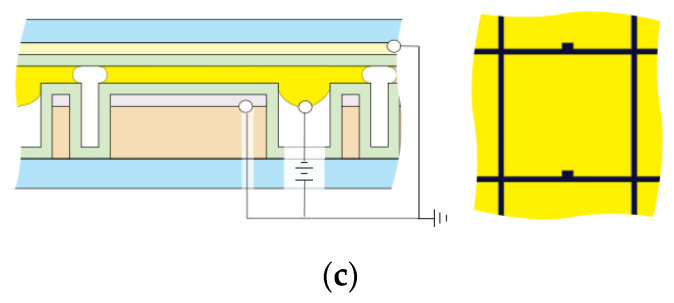
Three-dimensional honey-comb pixel structure and display principle for oil spreading and storing (**a**) honey-comb oil reservoir (**b**) oil-spreading for pixel opening (**c**) oil-storing for pixel closing (Images are reproduced from reference [21] with the permission of Springer Nature).

Taking the 5 micron (um) thick oil film as an example, when the pixel size is less than 50 um, the opening rate will drop to 70%. If the pixel size is less than 40 um, the opening rate will drop to 50%. In order to achieve smaller pixels and a higher opening rate, a feasible method is to make the oil film thinner. However, the oil film cannot be very thin since a thin oil film will reduce the illuminating contrast. The design of the “oil reserve structure” realizes the storage of the oil inks and is free of the limitations of the oil film thickness. Consequently, this effectively avoids the occurrence of the oil flow over the wall (overflows). As a result, this effectively prevents the optical defects. In addition, the oil grooves potentially limit oil movement which makes bi-stable electrowetting display possible [11]. In 2009, Heikenfeld et al. [21] developed a three-dimensional electrowetting display, as shown in Figure 9. The honey-comb like structure enables oil spreading and storing. When there is no electric signal, the color ink collects in the storage tank. The transparent oil layer is laid on the surface of the hydrophobic insulation layer, the light reflects through the oil layer and displays as white. When the voltage is applied, the ink is distributed to the surface of the hydrophobic insulation layer due to the effect of electrowetting. In the meantime, the oil phase originally laid on the display surface is pushed to the conduit, the light reflects and displays the color of the ink. The structure is good for the storage of the inks and increases the opening rate of the pixels, therefore improving the reflectivity.

**Figure 10:**
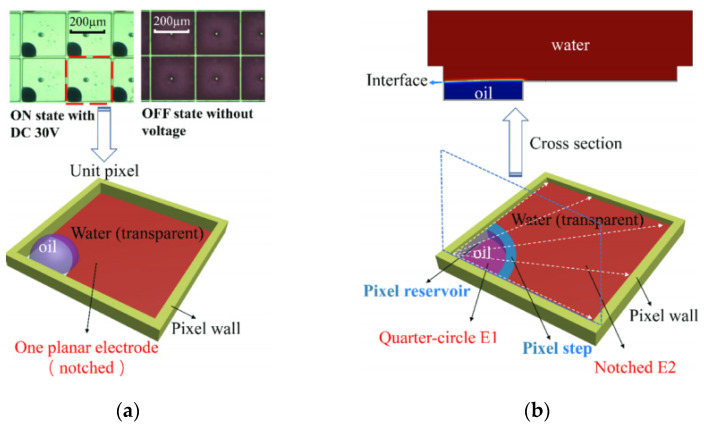
Illustration of the bi-stable “groove” EWD pixel structure (**a**) On-state “planner” pixel structure (**b**) On-state bi-stable “groove” pixel structure. (Images are reproduced from reference [22] with the permission of American Chemical Society).

In addition, the “oil reserve structure” was also applied on a conventional square display panel [22], as shown in Figure 10. This structure enables good control over the movement of the ink through the asymmetric distribution of the electric field, without affecting the visual effect. However, further improvements are required to resolve the issue related to the groove structure producing a local pinning force which has a certain impact on the reversibility of the ink movement.

## 5. Electrical Driving: Optimization of Opening Behavior and Greyscale Control

A good optical performance of an electrowetting display is realized by controlling the movement of ink under optimized driving signals. When the initial voltage is very low, the ink area is basically invariable. However, the voltage should not be too high otherwise it will cause the oil film to break into a number of small oil droplets, which reduces the display area. In addition, it can easily penetrate the hydrophobic insulation layer and damage the pixel unit. 

In order to display different gray scales in an electrowetting display, a voltage sequence must be applied in order to control the gray scale. This voltage sequence is related to the driving waveform. The driving waveforms commonly used in an electrowetting display are pulse width modulation (PWM) [23] and amplitude modulation (AM) [16]. However, both of these methods have drawbacks. The high frame rate of conventional PWM limits the gray level and causes large visual oscillations. The disadvantage of AM method is mainly attributed to the hysteresis effect.

**Figure 11:**
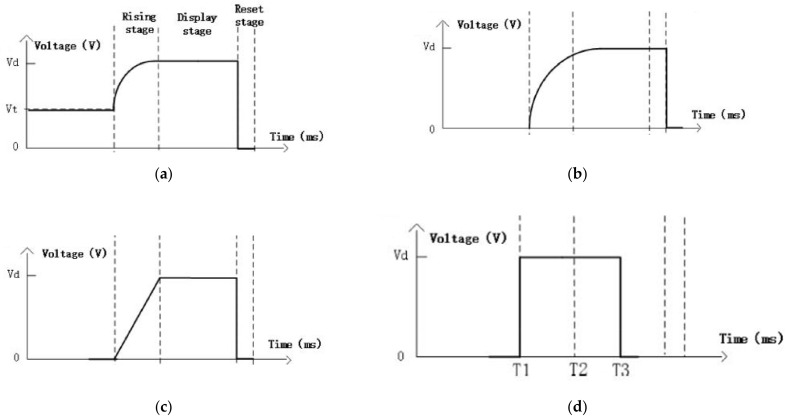
Modified pulse width modulation (PWM) driving waveforms (**a**) sinusoidal driving waveform after optimization (**b**) sinusoidal driving waveform (**c**) ramp driving waveform (**d**) square wave driving waveform. (Images are reproduced from reference [24] with the permission of Springer Japan).

A more functional driving waveform is based on the modified PWM square waves, as shown in Figure 11 [24]. The faster rising rate results in a shorter ink rearrangement time after oil film rupture [25]. When a sharp driving waveform is applied, the oil film breaks into oil drops which move to different corners; a process called oil splitting [15]. Slow waveforms, such as sinusoidal waveforms (as shown in Figure 11b), on the other hand, can effectively reduce the oil splitting and slowly open the pixel. However, the rising time is long, which limits the response rate. Therefore, the rising voltage waveform—as shown in Figure 11a—that is employed to start from the threshold voltage and rise to the pixel opening voltage in the form of a sine wave, not only improves the opening rate, but also reduces the oil splitting effect.

**Figure 12:**
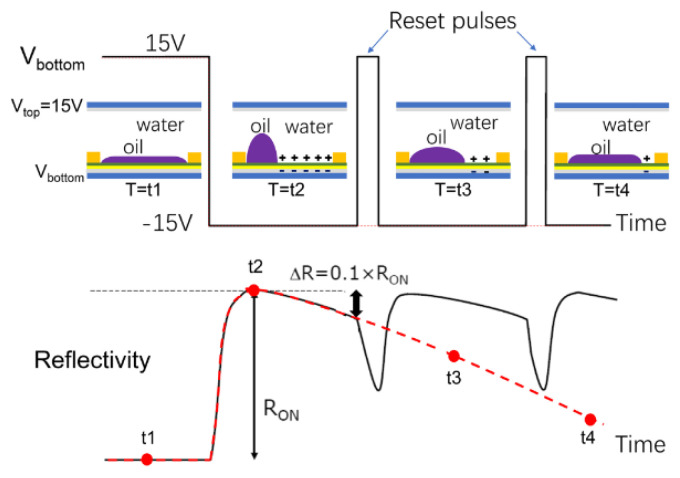
The voltage drive waveform of periodic reset and the change of reflectivity. (Images are reproduced from reference [26] with the permission of Wiley-Blackwell).

When a voltage is applied, the oil film opens and the oil ink is pushed towards the corner of the pixel. After a certain period of time, some ions from the liquid electrolyte enter the insulation layer and are trapped, thus causing ink “backflows” [26]. The experimental results showed that when an alternating current voltage is applied, the captured charge can be repeatedly released and recaptured [23]. Therefore, the charge capture behavior can be controlled by applying the alternating current signal so as to limit the ink backflow effect and improve the display stability. As shown in Figure 12, an initial voltage of 15 V is applied to both upper and lower plates, the voltage difference is 0 and the oil film remains in place. When the bottom voltage changes to −15 V the pixels slowly open to show the state at T2, and the ink shrinks to the corner of the pixel. At T3, due to the charge capture, the oil droplets gradually flow back. If the reflectivity is reduced to one tenth of the maximum value, the voltage between the plates is set to 15 V. In this way, the trapped charges are neutralized. Once the signal is set to −15 V, the reflectivity will rise again. A periodic reset will ensure a stable and high reflectivity. 

To provide a brief discussion of the amplitude-and-frequency modulation of the electric signal, the chosen voltage and frequency should be able to prevent the display defects (splitting, overflow and backflow) in order to improve the display performance and optical stability. Therefore, the initial voltage potential is set to a value just below the rising threshold and the applied voltage is slowly increased in the form of a sinusoidal wave. This will both improve the opening rate and reduce ink splitting and oil overflows to the adjacent pixels. The frequency of the periodic square waves is also fitted to prevent the phenomenon of ink backflow so that the pixel can maintain a constant optical reflectivity. 

## 6. Demonstration Displays and Color Reproduction

### 6.1. Color Mixing

**Figure 13:**
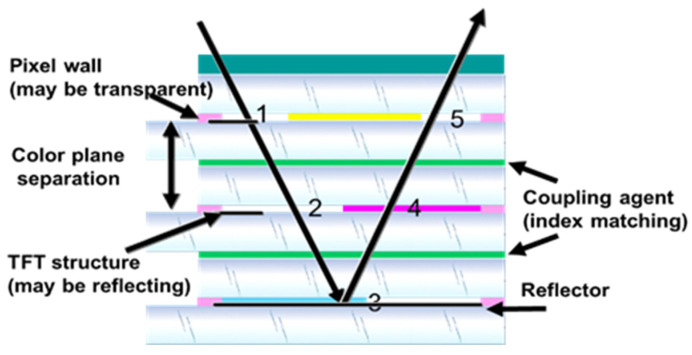
Parameters influencing parallax. (Images are reproduced from reference [27]).

The color system for this display is based on a cyan, magenta, yellow subtractive color system. This means that three layers of color have to be assembled and separately driven. Therefore, displays have to be aligned accurately, substrate thickness has to be minimized to limit parallax (Figure 13) and light losses have to be minimized in order to achieve brilliant colors.

### 6.2. System

The target for the display demonstration is to—as much as possible—use “standard” driving equipment. In order to achieve this, the image source of the demonstrated panel consists of a standard Android system, providing standard RGB output to drive the display panels.

The adaptation is surprisingly easy, once it is realized that cyan is the complement of red, magenta is the complement of green, and yellow is the complement of blue. Therefore, by driving the cyan panel with the red signal, the signal generates exactly the right response (less “cyan” means more “red”).

Of course, some adaptation may be necessary for grayscale spacing, but this is relatively simple, and can be done by adjusting the greyscale “gamma” value. Apart from this, the system has to accommodate for the peculiarities of the electrowetting system, making sure the charge rate of the pixel does not exceed a certain limit value, as described in previous sections. Furthermore, the system makes use of the field response and low leakage current properties of the display, making it possible to refresh a still image with long intervals, thereby minimizing the power.

## 7. Conclusions

**Figure 14:**
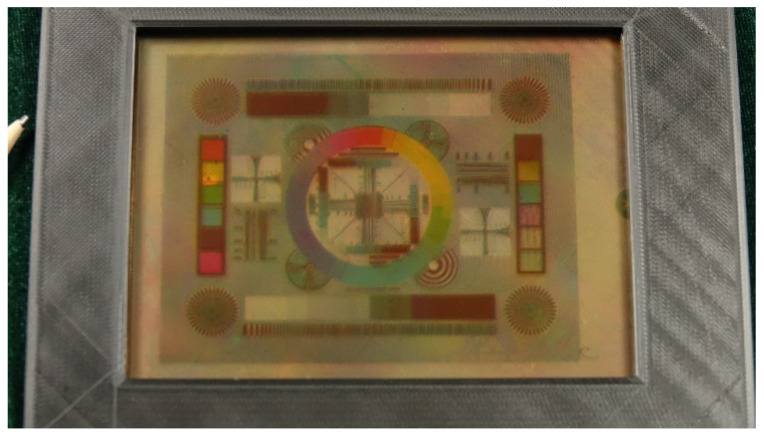
Prototype 5.8” full color reflective video speed electrowetting display.

The electrowetting display theory, technological development of dielectric layers, pixel structure designs, electric signal modulation and the current progress made in terms of color display are reviewed. The physical mechanism of electrowetting was first introduced and the failure mode of the dielectric layer was analyzed. Then, the roles of the pixel structure and driving mechanism in solving display defects were discussed. The “asymmetric breaking structure” is helpful to control the opening direction of the oil film, which ensures the consistency of the pixel opening. The use of the “oil reserve structure” is good for ink storage and control of ink movement, which greatly prevents display drawbacks, such as backflow and overflow. The bi-stable structural features also reduce energy consumption and extend the service time. The driving scheme is adopted by a two-step method. Firstly, a threshold voltage is set in the preparation stage, and then it slowly rises to the threshold for opening the oil film in a gradual waveform. Secondly, after maintaining this voltage difference for a period of time, periodic alternating signal regulation is carried out in time. This waveform effectively prevents the oil film splitting into several smaller oil droplets and also avoids the oil backflows caused by the capture of charges in the dielectrics. Therefore, future research can explore the fabrication of multi-layer dielectrics, the design of hierarchical pixel structures and the optimization of driving schemes, which are expected to resolve the current limitations in EWD and improve display performance. Lastly, a full color reflective video speed electrowetting display panel (Figure 14) is proposed for future applications.
